# Impact of Culture Medium on Cellular Interactions in *in vitro* Co-culture Systems

**DOI:** 10.3389/fbioe.2020.00911

**Published:** 2020-08-04

**Authors:** Michelle A. M. Vis, Keita Ito, Sandra Hofmann

**Affiliations:** ^1^Orthopaedic Biomechanics, Department of Biomedical Engineering, Eindhoven University of Technology, Eindhoven, Netherlands; ^2^Institute for Complex Molecular Systems, Eindhoven University of Technology, Eindhoven, Netherlands

**Keywords:** co-cultures, *in vitro* models, culture medium, medium exchange, biosensors

## Abstract

Co-culturing of cells in *in vitro* tissue models is widely used to study how they interact with each other. These models serve to represent a variety of processes in the human body such as development, homeostasis, regeneration, and disease. The success of a co-culture is dependent on a large number of factors which makes it a complex and ambiguous task. This review article addresses co-culturing challenges regarding the cell culture medium used in these models, in particular concerning medium composition, volume, and exchange. The effect of medium exchange on cells is often an overlooked topic but particularly important when cell communication via soluble factors and extracellular vesicles, the so-called cell secretome (CS) is being studied. Culture medium is regularly exchanged to supply new nutrients and to eliminate waste products produced by the cells. By removing medium, important CSs are also removed. After every medium change, the cells must thus restore their auto- and paracrine communication through these CSs. This review article will also discuss the possibility to integrate biosensors into co-cultures, in particular to provide real-time information regarding media composition. Overall, the manner in which culture medium is currently used will be re-evaluated. Provided examples will be on the subject of bone tissue engineering.

## Introduction: *In Vitro* Tissue Models

Before culturing of cells was possible, animals were used to study human physiology and pathophysiology, in particular in medical and pharmaceutical industries (Russell and Burch, 1959). Animal models frequently failed to capture important facets of human physiology and pathophysiology and thus failed to mimic true human responses (Holmes et al., 2009). The possibility to culture human cells increased our insight into healthy and diseased states of the human body (Thomson et al., 1998; Holloway et al., 2019). First, cells were cultured in monolayers which in some cases lacked the complexity needed to study diseases and responses to drugs thoroughly (Esch et al., 2014; Fu et al., 2017; Zhang et al., 2019). Three-dimensional (3D) models enabled the creation of a cell environment closer to the natural microenvironment, increasing the potential to predict physiological responses and also increasing complexity. For example, different 3D *in vitro* models to study osteocytes were established recently, mimicking their native environment and showing superior morphology and behavior compared to monolayer cultures, enabling future development of human disease models (Zhang et al., 2019).

The approach for the design of *in vitro* tissue models originates from tissue engineering (TE; Langer and Vacanti, 1993; Caddeo et al., 2017). TE combines cells, scaffolds, growth factors and mechanical stimuli to create tissues *in vitro*. Traditionally, TE has focused on the creation of tissue grafts for implantation. More recently, TE has been applied to develop *in vitro* tissue models. In contrast to tissue grafts that need clinically relevant sizes of engineered tissue, *in vitro* models aim to resemble the smallest functional unit of a tissue. Such *in vitro* models show potential to study processes of the human body such as development (Robin et al., 2016), homeostasis (Rossi et al., 2018), regeneration (Guzmán et al., 2014), and disease (Salamanna et al., 2016).

The development of 3D human *in vitro* models depends on the ability to partially recreate the complexity of the native microenvironment that defines cues (physical, chemical, and biological) for cell function, proliferation, and differentiation (Holmes et al., 2009). The challenge is to define the aspects of the microenvironment which are important in order to engineer the smallest functional unit that captures the interaction between key cues in the cell system which it controls (Holmes et al., 2009). Research has shifted toward improving *in vitro* models by increasing their complexity in order to understand how mature intricate tissues form (Holloway et al., 2019). An increase in complexity can be accomplished by culturing different cell types together in one culture, called co-culturing.

## Co-Cultures With the Application for *In Vitro* Tissue Models

Co-culturing of cells is widely used to study interactions between cell populations in many fields including (but not limited to) synthetic biology (Goers et al., 2014), ecology (Jessup et al., 2004), TE both 2D and 3D (Liu et al., 2015; Paschos et al., 2015), and multi-organ microphysiological systems (Wang et al., 2017). Models have been developed for a variety of tissues such as lung (Strikoudis et al., 2019), intestine (Jalili-Firoozinezhad et al., 2019), kidney (Takasato and Little, 2017), bone (Rossi et al., 2018), embryo (Saadeldin et al., 2014), ovary (Saadeldin et al., 2015), neuron-glia (Skaper and Facci, 2018), and liver (Coll et al., 2018). Co-cultures can be used to represent both physiological and pathological tissue states. Ideally, human, or even patient-specific cells are used to create cellular environments that are more representative for humans rather than animal derived cells (Caddeo et al., 2017). Most co-culture studies involve two cell types, owing to an increased complexity in establishing a stable system when more cell types are involved (Goers et al., 2014). There are also studies reporting the use of three (Venter and Niesler, 2018; Churm et al., 2019; Lin et al., 2019) or even four cell types (Zhang et al., 2009; DesRochers et al., 2015).

**FIGURE 1:**
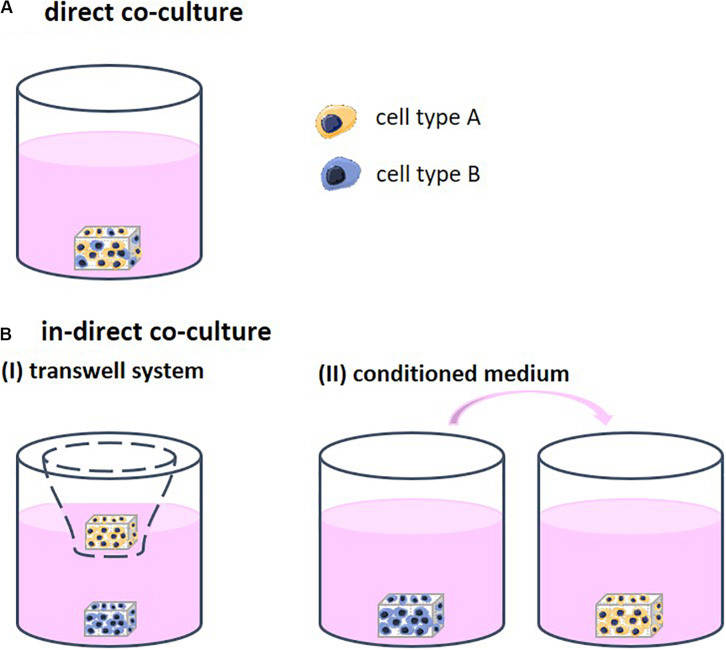
**(A)** Direct co-cultures facilitate physical contact between the different cell types which allows for communication though their surface receptors. **(B)** Indirect co-cultures incorporate a physical separation between cell types only allowing for communication via cell secretomes. **(I)** Physical separation in the form of a transwell system using a semi-permeable membrane. **(II)** Conditioned medium is first collected from one cell type and then transferred to the other cell type. The medium contains the cell secretome of the first cell type, which then affects the second cell type.

Different strategies to co-culture cells in 3D exist, each allowing for a different degree of contact between the cell types. Through this contact, the cells are able to stimulate each other. Direct co-cultures facilitate physical contact between the different cell types which allows for communication though their surface receptors and gap junctions, defined as juxtacrine communication (Figure 1A). Indirect co-cultures incorporate a physical separation between cell types, such as a semi-permeable membrane in the form of a transwell system, only enabling signaling via the cell secretome (CS; Figures 1B-I). In addition, in indirect co-cultures, conditioned medium is frequently used (Figures 1B-II). Medium is first used for culturing one cell type and then transferred to the second cell type. The medium contains the CS of the first cell type, which then affects the second cell type. Conditioned medium contains numerous CSs that may positively and/or negatively regulate cell behavior (Katagiri et al., 2017). The mechanisms that support the effect of these CSs remain insufficiently defined and are highly dependent on the cell source (Marolt Presen et al., 2019).

Cell secretomes ensure cell-cell communication and comprise of soluble factors and cell-derived membranous structures. These so-called extracellular vesicles (EVs), are nanosized particles (exosomes, 30–100 nm; microvesicles, 50–2000 nm; Martins et al., 2016) that transfer proteins, bioactive lipids between cells. Moreover, EVs are also capable of transferring RNA between cells, called exosomal RNA or esRNA (Lotvall and Valadi, 2007; Valadi et al., 2007). EVs are present in biological fluids and are involved in multiple physiological and pathological processes (van Niel et al., 2018). For example, EVs derived from osteogenically committed mesenchymal stromal cells were shown to induce osteogenic commitment of homotypic cells without further supplementation (Martins et al., 2016). EVs are widely studied for their potential as a cell-free therapeutic method for regeneration of numerous tissue types. Subsequently, EVs might be used to study cellular interactions *in vitro* omitting the requirement for a co-culture experiment and thus overcoming co-culture challenges. The biggest challenge of using EVs lies within the development of purification and characterization protocols (Lee et al., 2019).

Overall, co-cultures are versatile models to create cellular environments in which interactions between different cell types can be studied *in vitro*. These interactions can take place by direct contact and by exchange of soluble factors and EVs. This review article will focus on steps that can guide optimization of medium composition and volume in co-cultures with a particular focus on cell communication via the CS.

## General Considerations Regarding Culture Medium in Co-Cultures

### Selecting Culture Medium Composition

In cell culturing, culture medium is added to nourish the cells. Culture medium is a liquid nutritive substance consisting of a mixture of base medium, serum, and regulating factors. Firstly, base medium fills the nutritional requirements of the cells. The first base medium was developed in 1959 and was defined as the Minimal Essential Medium (MEM), including 13 amino acids, 8 vitamins, 6 ionic species, and glucose (Eagle, 1959). Secondly, serum, such as fetal bovine serum (FBS) contains important basic proteins including growth factors and hormones for maintaining cell survival, growth, and proliferation (Gstraunthaler, 2003). FBS is a complex and natural mixture that is extracted from fetal blood. The use of FBS is controversial due to quality and reproducibility issues as well as animal welfare concerns which is elaborately reviewed elsewhere (van der Valk et al., 2018). Thirdly, regulating factors such as growth factors are added to the medium to guide specific and desired cell behavior such as proliferation and differentiation into a particular cell lineage. These factors are key in cell cultures as they predominantly determine cell fate. Establishing a functional and precise mixture of these culture medium ingredients is of great importance for creating *in vitro* tissue models.

**FIGURE 2:**
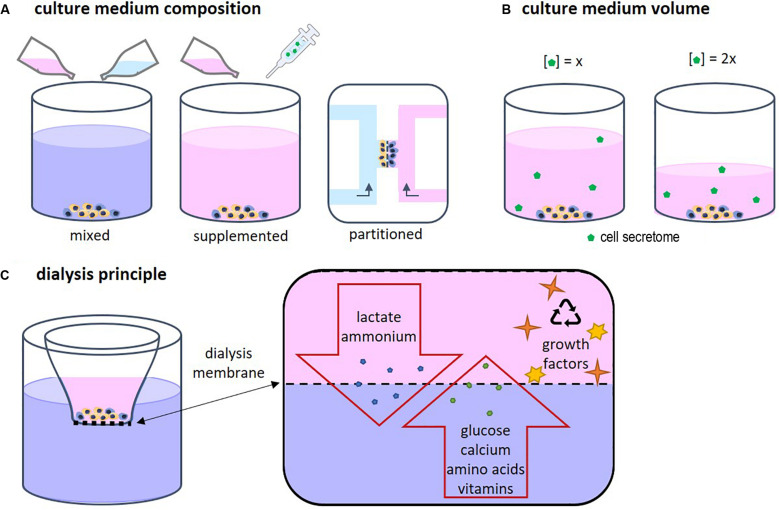
**(A)** Several approaches for co-culture medium optimization have been tested, such as mixed medium, supplemented medium, and fluidically partitioned culture environments. **(B)** The culture medium volume has an influence on the concentration of the cell secretome. **(C)** The principle of dialysis of culture medium relies on the size of the components in the medium. Depending on the chosen MW cut-off, the dialysis membrane allows for exchange of low MW proteins, amino acids, vitamins, lactate, and ammonium while high MW components such as growth factors, are retained in cell culture insert.

Each cell type has specific needs according to its function and requires a corresponding specific medium composition. When two or more different cell types are cultured together, choosing the right medium becomes a challenge (Goers et al., 2014). Several approaches are possible, such as mixed medium, supplemented medium and partitioned culture environments (Figure 2A). In a mixed medium, the medium of all used cell types is combined, possibly in different ratios. With this method, the original medium supplements might interfere with the other cell type, which is particularly important when culturing progenitor cells as these cells yet have to differentiate into the desired cell type. For instance, in a co-culture of precursors of osteoblasts and osteoclasts, the osteogenic supplements dexamethasone and β-glycerophosphate are needed for osteoblast differentiation and maturation, while these supplements have been shown to inhibit monocyte differentiation into osteoclasts (Haynesworth et al., 1996; Takeyama et al., 2001; Langenbach and Handschel, 2013). An optimum dosage of supplements has to be found in order to obtain both functional osteoblast and functional osteoclasts. Another approach could be to use a general base medium, supplemented with the soluble factors that stimulate both cell types without negatively affecting either of them (Zhu et al., 2018). This method makes it possible to modulate the medium more specifically than by just mixing two media types. The disadvantage is that it is time consuming to find suitable supplements and to optimize the combination. Additionally, a culture method that enables two physically partitioned medium flows can be used. In this way, both cell types receive their specific medium while cell-cell contact is still possible (Robertson et al., 2014). However, this is a complicated and precise method that can mostly be performed in 2D and for certain cell types.

In multi-organ microphysiological systems, the challenge of finding the right medium is even more difficult as a variety of cell types may each have their own optimal medium and supplements. For example, in a device combining liver, lung, kidney, and adipose tissue, it was shown that addition of transforming growth factor β1 (TGF-β1) supported the growth of lung cells but inhibited the growth of liver cells (Zhang et al., 2009). They overcame this by using gelatin microspheres that released TGF-β1 locally to support the lung compartment while in the circulation, low TGF-β1 levels could be maintained (Zhang et al., 2009).

Just as important, one cell type in a co-culture naturally provides CSs that influence the other cell type. As a result, medium supplements might have to be altered in concentration or might be fully omitted (Zhu et al., 2018). For example, osteoclasts in mono-culture are derived from mononuclear cells by addition of macrophage colony-stimulating factor and receptor activator of nuclear factor kappa-B ligand. Both molecules are naturally produced by osteoblasts (Boyce and Xing, 2007). Thus, in a co-culture with osteoblasts, no additional cytokines may be needed for osteoclast formation (Schulze et al., 2018; Zhu et al., 2018).

Medium optimization is crucial but is laborious and time-consuming because of the enormous number of possible combinations. Parallel assays using micro/nano-scale devices hold great promise for evaluation and optimization of a multitude of options (Sasaki et al., 2016). For example, a sensitive platform for optimum culture media investigation was developed in which image-based profiling was combined with microdevices to achieve high-throughput evaluation of culture medium conditions (Sasaki et al., 2016). Advances in this field could be of great value to ease the inconvenience of medium optimization.

### The Effect of Culture Medium Volume

Medium volume is of importance as a higher volume leads to lower concentrations of the CS (Figure 2B). In bone cell cultures, osteoblastic mineral deposition and fusion of osteoclast-precursors into osteoclasts were shown to be dependent on the medium volume (Yoshimura et al., 2017). When culturing cells, often the medium volume suggested by the manufacturer of culture plastics is used. However, this volume is not optimized for specific cell types. For example, low culture medium volumes not only have been shown to be beneficial for culturing cell types such as neuron-like cells (Shimomura et al., 2016) and adipose derived mesenchymal stem cells (Simão et al., 2019), they are also more economical. On the other hand, some culturing conditions, for example in bioreactors, might require minimal volumes to operate, which makes volume optimization impracticable. In these cases, it should be recognized that the medium volume may impact a variety of cell culture aspects (Yoshimura et al., 2017).

Medium volume is influenced by cell culture aspects such as nutrient supply, dilution, or concentration of waste products and metabolites, and changes in oxygen level (Zhu et al., 2018). Studies have demonstrated that the oxygen concentration in medium decreases with increasing medium depth, leading to altered cell growth characteristics (Oze et al., 2012; Place et al., 2017). Moreover, it has been recognized that cell proliferation and differentiation are largely influenced by the concentration of CSs (Yoshimura et al., 2017). With different medium volumes these CSs become either more or less concentrated resulting in faster or slower proliferation and differentiation of these cells. Thus, cells might function differently when cultured in different medium volumes. Again, optimization is key but laborious and, in some cases, even impracticable. Therefore, one should be aware of the effects of medium volume. Certainly, when unexplainable results are encountered and when protocols are adjusted to up- or down-scale experiments.

## The Effect of Medium Exchange on Cell-Cell Interactions

### Waste Accumulation Problem

Medium is exchanged regularly to maintain nutrients and growth factors consumed by the cells and to eliminate waste products produced by the cells. Mammalian cells use glucose for energy and produce lactate as a metabolite (Ozturk et al., 1997). *In vitro*, every cell type needs a narrow pH range within 0.2 to 0.4 pH units of its optimum to grow (Paul, 1975). The production of lactic acid should not exceed the buffering capacity of the medium, because lowering the pH can inhibit cell growth (Zielke et al., 1980; Glacken et al., 1986; Ozturk et al., 1992, 1997). Also, high ammonium concentrations as a by-product of glutamine catabolism can be toxic to cells causing cytosol vacuolization and subsequent cell death (Glacken et al., 1986; Slivac et al., 2010). Exchanging the medium prevents these waste product accumulation effects.

However, after every medium exchange, also the CS is removed, and the cells must make a new effort to restore their communication by producing fresh molecules. This effort could negatively influence their behavior, not representing their natural state. The influence of medium exchange was for example investigated by measuring actin microfilament structure directly before and after medium exchange (Krüger-Genge et al., 2015). Medium exchange led to a rapid disturbance of stress fiber formation and disconnection of cell-cell contacts. Frequent medium exchange is also economically disadvantageous as medium can contain expensive additives such as growth factors and animal serum (Glacken et al., 1986). However, medium exchange cannot be prevented as nutrient deprivation and waste accumulation would lead to inevitable cell death.

### Systems for Culture Medium Re-use

Driven by economical motives, re-use of medium was first described in 1977 by adding fresh nutrient supplements to used medium (Mizrahi and Avihoo, 1977). However, due to the accumulation of waste products, the medium could only be re-used once. A second re-use caused cell death. To overcome this issue, other cell culture systems were developed in which medium was dialyzed to remove waste products. In addition, dialysis could be used to harvest cell products such as antibodies (Adamson et al., 1983). The principle of dialysis relies on the exclusion of molecules based on their size (Figure 2C). Fresh medium contains low molecular weight (MW) molecules such as nutrients, amino acids, and vitamins. Depending on the chosen MW cut-off, the dialysis membrane allows for exchange of those molecules. In this way waste products can diffuse out of the culture medium while nutrients and vitamins diffuse back in. High MW components such as growth factors are retained in the medium compartment.

The first dialysis system cultures were rather complex and large. For example, a bioreactor was developed using a 5 liter medium vessel coupled to a 2 liter perfusion system (Büntemeyer et al., 1992). Several fluid streams were connected to control waste removal, medium recycling, and nutrient supply. For the elimination of toxic waste products, a hollow fiber microfiltration system was used while nutrients were supplied by adding concentrated solutions. Most previous studies focused on mass production, generally using large scale reactors (Chen et al., 2011; Nath et al., 2017). Recently, a simpler dialysis culture system was presented that does not require the use of pumps and vessels (Shinohara et al., 2019). A deep well culture plate including an insert with a dialysis membrane was used (Figure 2C). Successful and continuous glucose supply and lactate removal through the dialysis membrane were shown. The retaining of cytokines and autocrine factor enabled to promote endodermal differentiation of induced pluripotent stem cells (iPSCs) without daily cytokine addition. This dialysis system for re-using culture medium still is not frequently applied and mainly used for proliferation and differentiation processes of (costly) iPSCs studies (Côme et al., 2008; Nath et al., 2017; Shinohara et al., 2019). Use of these dialysis systems in other cell culturing fields requires optimization. For example, the size of medium components should be known and taken into account as high MW proteins, which are also found in FBS, will not be able to cross the dialysis membrane. In our opinion, medium dialysis could not only reduce culture costs, it could also contribute to a more physiological environment for cell proliferation and differentiation. This would especially be true for co-cultures where the interaction between different cell types is investigated, by retaining the communication factors produced by the cells.

## Implementation of Biosensors

Combining biology and technology advances cell culturing at a rapid pace. Addition of biosensors to cell cultures is one of these beneficial combinations. Biosensors show potential for monitoring of the microenvironments in *in vitro* systems and aim at providing real-time information regarding cell viability, growth and metabolism (Pereira Rodrigues et al., 2008; Modarres et al., 2018; Young et al., 2019). For example, on-line measurement of dissolved oxygen was applied for medium optimization of mammalian cell cultures (Deshpande et al., 2004). An oxygen sensor immobilized at the bottom of each well in a 96 wells plate was successfully used to optimize the concentration of glucose, glutamine and inorganic salts. This method was highly cost effective and time efficient, automatically analyzing many samples in one go in small medium volumes.

In order to maintain cell viability, experimental validity and reproducibility, it is essential that metabolite levels are maintained within physiological limits (Place et al., 2017). For example, fluctuations in oxygen and glucose concentration can affect cell growth, differentiation and signaling (Place et al., 2017). Multiplexed sensing, recording, and processing of real-time data could provide novel insights into the optimal nutrients and culture conditions needed to grow cells (Young et al., 2019). Furthermore, real-time data analytics can be used to respond to changes in culture conditions in a closed feedback loop, adjusting inputs to obtain desired results (Young et al., 2019). Sensors could provide help in determining the status of the cell culture. For example, medium composition can be tracked for the CS as a stem cell differentiates to determine how differentiation is progressing. Accordingly, growth factors can be removed or added to encourage further differentiation (Young et al., 2019).

It needs to be mentioned that while the technology is available, not many user-friendly and affordable techniques have been implemented into *in vitro* tissue cultures. Particularly techniques developed for continuous detection of biomolecules at low physiological concentrations require thorough understanding of electrochemistry, electrical engineering, and/or optics. Implementation will require a closer collaboration between researchers of different fields, willing to combine each other’s expertise, requirements, and possibilities.

## Conclusion

Investigating cell-cell interactions through CSs requires complex tissue cultures where different cell types are being co-cultured. Co-culturing asks for a highly specific environment meeting the requirements of all involved cell types and therefore requires a great deal of optimizing. Advances in this field bring us closer to *in vitro* models that can be used to study physiological and pathological cell-cell interactions and will allow for the development of drugs that interact with cells. We highly recommend to reconsider today’s method of complete medium exchange to provide a more physiological environment to the cells. Combining current *in vitro* culturing techniques with existing technological inventions such as dialysis and biosensors could lead toward the goal of developing more complex, reproducible, nature-like *in vitro* tissue models.

## Author Contributions

MV and SH contributed to the conception of the review. MV wrote the first draft of the manuscript. All authors contributed to manuscript revision, read, and approved the submitted version.

## Conflict of Interest

The authors declare that the research was conducted in the absence of any commercial or financial relationships that could be construed as a potential conflict of interest.
